# Primary CNS vasculitis: insights into clinical, neuropathological, and neuroradiological characteristics

**DOI:** 10.3389/fneur.2024.1363985

**Published:** 2024-04-08

**Authors:** Tahani Saker Sheikh, Ayal Rozenberg, Goni Merhav, Alla Shifrin, Polina Stein, Shahar Shelly

**Affiliations:** ^1^Department of Neurology, Rambam Medical Center, Haifa, Israel; ^2^Rappaport Faculty of Medicine, Technion-Israel Institute of Technology, Haifa, Israel; ^3^Department of Neuropathology, Rambam Medical Center, Haifa, Israel; ^4^Institute of Pathology, Rambam Medical Center, Haifa, Israel; ^5^Department of Neurology, Mayo Clinic, Rochester, MN, United States

**Keywords:** PCNSV, neuropathology, neuroradiologic diagnosis, atypical cases, immunomodulatory treatments

## Abstract

**Background and objectives:**

Primary CNS vasculitis (PCNSV) is a rare inflammatory disorder that affects the blood vessels of the central nervous system (CNS). We aimed to analyze the neurological presentations, clinical follow-up, and long-term outcomes of patients with primary central nervous system vasculitis.

**Methods:**

We conducted a retrospective analysis of medical records to assess the neurological presentation, rate of remission, and functional status at the last follow-up in patients with primary central nervous system vasculitis seen in our center in the last 13 years (2010–2023).

**Results:**

We identified five patients, whose median age at symptom onset was 31 years (range: 15–41 years), including four male individuals (80%) from Muslim Arab (*n* = 4) and Ashkenazi Jewish (*n* = 1) backgrounds. Symptoms persisted for a median of 36 weeks (range: 3 weeks to 4 years) before diagnosis, with one case exceeding 3 years. Follow-up lasted a median of 56 months (range: 20–161 months). Clinical symptoms varied, presenting unilateral weaknesses (*n* = 2), cognitive and gait abnormality (*n* = 1), headaches (*n* = 1), and epileptic seizures (*n* = 1). MRI scans revealed abnormalities in the basal ganglia, corona radiata, parietal, and frontal lobes, showing hemorrhage, vasogenic edema, restricted diffusion, and enhancement post-gadolinium. All patients reported progressive holocephalic headaches and cognitive changes with overall progressive symptoms. Initial neurological examinations revealed abnormalities in all patients and included one or more of the following: cognitive or visual impairment (*n* = 2), seizures (*n* = 1), and unilateral UMN signs (*n* = 2) at the initial neurological examination, all but one patient required walking aids including (cane 2, wheelchair, bedridden 1). Patients were stable (*n* = 2), deteriorated (*n* = 1), or improved (*n* = 2). Following treatment, two patients still required ambulatory aids, with one using a cane and the other using a wheelchair, while the remaining three did not require any ambulatory aids.

**Discussion:**

The study on PCNSV highlights varied symptoms and diagnostic challenges, including delayed diagnosis and a spectrum of neurological issues from cognitive impairments to seizures. Brain biopsies showed lymphocytic infiltration, thrombi, and necrosis. Immunotherapy significantly improved clinical and radiological outcomes. Over 56 months of follow-up, outcomes varied from stability and deterioration to improvement.

## Introduction

CNS vasculitis is a disorder characterized by inflammation and destruction of blood vessels within the brain, spinal cord, and meninges. The condition is multifaceted, and primary angiitis of the CNS (PACNS) is the preferred terminology for vasculitis solely affecting the CNS. Involvement of the CNS is often associated with systemic vasculitis that affects medium-sized and small-sized blood vessels (1–3). In patients with PACNS, the most common symptoms include headache, encephalopathy, and ischemic or hemorrhagic stroke. These symptoms can result from the inflammatory process that distracts and occludes the blood vessels in the brain, spinal cord, and meninges, which are characteristic of the disease. Other symptoms that may be present include seizures, cognitive impairment, and psychiatric symptoms (1–4). The presentation and severity of symptoms can vary depending on the location and extent of the vasculitis (1–6). The two former types are subacute and insidious, which account for a protracted duration between symptom onset and diagnosis of up to 6 months. Systemic symptoms such as fever, weight loss, and night sweats are relatively uncommon, occurring in less than 20% of cases or elevated erythrocyte sedimentation rate (ESR) in 25% of cases (2, 3).

Imaging and laboratory data can aid diagnosis using brain MRI, angiography, and CSF examination. Brain MRI abnormalities are reported in up to 96% of patients (5, 7, 8). Angiography is another leading tool to help in the diagnosis with an estimated sensitivity of 50 to 90% (9–11). Typical MRI findings show multifocal areas of signal intensity in both the white and gray matter, the presence of gadolinium-enhancing lesions, and evidence of both acute and chronic strokes. Angiography findings often show segmental narrowing, beading, or dilation of vessels, although sensitivity can vary. Typical CSF findings include mild lymphocytic pleocytosis, elevated protein levels, and occasionally oligoclonal bands, indicative of an inflammatory process. Brain tissue biopsy demonstrating angiitis remains the gold standard. Calabrese and Mallek proposed diagnostic criteria for PCNSV that are still valid (12, 13), including historical or clinical findings of an acquired neurological deficit of unknown origin after a thorough initial basic assessment, cerebral angiogram with classic features of vasculitis, or a CNS biopsy showing vasculitis; and no evidence of systemic vasculitis or any other disorder. PCNSV is distinguished from systemic vasculitis by ruling out systemic symptoms, conducting extensive laboratory work-up including CRP, ESR, and serology for autoimmune connective tissue diseases, and performing PET-CT to rule out the involvement of other subclinical organs in a systemic inflammatory process. As PCNSV may present with acute focal neurological signs fulfilling clinical criteria for acute stroke, administration of IV TPA is not well studied and could even be harmful, considering the high likelihood of secondary hemorrhagic conversion in such patients who present with ischemic stroke, the use of thrombolysis is controversial and should be regarded with extreme caution in the absence of other risk factors for stroke and should be regarded as a relative contraindication. Treatment should be initiated promptly with the drugs of choice, usually corticosteroids with cyclophosphamide. Immunosuppressants such as azathioprine, methotrexate, mycophenolate mofetil, or rituximab can also be used (14).

This study reviews the clinical features, treatment, and long-term outcomes of five patients with PACNS who were admitted to our center, providing further insight into the characteristics and management of this rare condition.

## Methods

This study was approved by the Institute of Medical Sciences Institutional Ethics Committee.

### Cohort selection

From January 2010 to July 2023, five patients at Rambam Medical Center (RMC) with definite diagnoses of PCNSV were included. Two neurologists verified all clinical data (S.S and T.S). Age of onset was defined as the age at which the first clinical features became apparent. We included adult patients (age ≥ 18 years) with a diagnosis of PCNSV who had a recent history or presence of an acquired unexplained neurological deficit, and all had evidence of vasculitis in a brain biopsy with changes characteristic of vasculitis. Excluded patients had evidence of systemic vasculitis or other PCNSV mimickers (neurosarcoid or neuro-Behcet’s disease), or they had findings explained by other causes.

Serological vasculitis markers, including CRP, ESR, ANA, anti-dsDNA, anti-CCP, p/c ANCA, anti-cardiolipin, anti-beta2 glycoprotein, SSA, and SSB, were tested in all patients. Chronic infectious etiologies were ruled out by testing serology for RA factor, VZV, HCV, HBV, HIV, CMV, West Nile virus, Q-fever, syphilis, Brucella and Bartonella, cryptococcal antigen, and negative two separate blood culture sets. Additionally, HLA-B51, RA factor, anti-SSA and SSB, cryoglobulins, and 2D echocardiography were performed based on the patient’s clinical profile.

### Imaging and histopathology studies

Brain MRI scans underwent a rigorous review and analysis by two neuroradiologists who examined them for various abnormalities including changes in the gray and white matter, hemorrhages, and lesions with enhancements in different regions of the CNS. They also assessed the images for arterial abnormalities such as steno-occlusive changes, irregularity, beaded pearls appearance, and aneurysms, as well as venous phase abnormalities such as dilatation and tortuosity of small veins, puddling and staining of contrast, and parenchymal venous phase abnormalities. All our patients underwent CTA examinations, while two patients underwent conventional angiography; these examinations did not reveal any vascular abnormalities.

All patients underwent brain biopsy, either targeted (sampled from regions of the brain that demonstrated abnormalities on imaging) or blinded (non-dominant frontal or temporal pole). Our priority was to perform a targeted biopsy. In one case, we performed a blinded biopsy due to the unsuitable location of the lesion for biopsy. They were reviewed and reported by the neuropathologist, and a PCNSV diagnosis required the presence of transmural inflammation of small- or medium-sized meningo-cortical blood vessels. It was further classified into granulomatous, lymphocytic, and necrotizing subtypes.

### Statistical analysis

Categorical variables were reported as proportions, whereas continuous variables as medians with interquartile range. Wherever applicable, differences in categorical variables were assessed using the chi-square or Fisher’s exact test, whereas continuous variables were assessed using the Mann–Whitney test. Univariate and stepwise multiple logistic regression was applied to find independent predictors of good functional outcome (mRS 0–2), and an adjusted odds ratio was calculated. *p*-value < 0.05 was considered statistically significant. All statistical analyses were performed using SPSS version 27.0.

## Results

### Demographic, clinical manifestation, and laboratory studies

We identified five cases of primary CNS vasculitis (PCNSV) admitted to our ward between 2010 and 2023. The demographic characteristics of these patients are presented in Table 1. The median age of symptom onset was 31 years (range: 15–41 years), with 4 male individuals (80%). Ethnic ancestries were Muslim Arabs (*n* = 4) and Ashkenazi Jews (*n* = 1). The median duration of symptoms until diagnosis was 36 weeks (range: 3 weeks to 4 years), with one patient diagnosed more than 3 years after symptom onset. Table 2 provides a synopsis of the clinical presentations. The median follow-up time was 56 months (range: 20–161 months).

**Table 1:** Demographic, clinical, and testing characteristics of PCNSV patients.

**Category**	
Sex, Male	4/5 (80%)
Median onset age, years	31
Median time to final follow-up, months	56
Median duration of symptoms, weeks	36 weeks
Median time to diagnosis, weeks	36 weeks
Sensory ataxia on examination, %	40%
Falls, %	80%
Use of gait aids, %	80%
Central sensory loss on examination, %	60%
Motor weakness on examination	40%
Increase deep tendon reflexes on examination, %	100%
Median CSF protein mg/dL	57 mg/dL
Lesion enhancement, axial (MRI) %	80%
Treatment responsiveness, %^1^	100%
Use ambulatory aids pre-Rx, %	80%
Use ambulatory aids post-Rx, %	40%

**Table 2:** Case-by-case demographic, clinical, and testing characteristics of PCNSV patients.

**Univariate analysis**	**Case 1**	**Case 2**	**Case 3**	**Case 4**	**Case 5**
Sex	M	M	M	F	M
Age	29	19	43	40	54
Median symptom onset age (IQR)	24	15	37	31	41
Age at diagnosis	25	19	38	31	41
Delay in diagnosis	8 months	4 years	1.2 year	1 Month	3 weeks
Initiation of treatment (time from first symptoms onset)	8 months	4 years	1.2 years	1 month	1 month
Headache	Yes	Yes	Yes	Yes	Yes
Cognitive impairment	No	Yes	Yes	No	Yes
Hemiparesis	Yes	Yes	Yes	No	No
Progressive course	Yes	Yes	Yes	Yes	Yes
Multiphasic course	No	Yes	Yes	Yes	Yes
Paraparesis	No	No	No	No	No
Ataxia	Yes	Yes	Yes	No	No
Seizure	No	No	No	No	Yes
Visual impairment	No	No	No	Yes	Yes
Neurological symptoms/signs	Subacute onset of distal right hand and leg weakness and headache.	Brainstem infarct 2019. 2023-acute onset of right-sided weakness and headache.	1-year progressive cognitive decline, spasticity, and walking difficulty with headache.	Rapidly progressive headache and decreased responsiveness.	Acute generalized seizures
Abnormal CSF protein	Not relevant	Not relevant	Yes (57 mg/dL)	Yes (84 mg/dL)	No (41)
CSF leukocytosis	Not relevant	Not relevant	Yes (15 LYM)	Yes (234)	No (6)
Spinal cord involvement by disease	Not relevant	Not relevant	No	No	No
Hemorrhages	No	Yes	No	No	Yes
Granulomatous PCNSV	No	No	No	Yes	No
Lymphocytic PCNSV	Yes	Yes	Yes	No	Yes
Treatment (initial)	Methylprednisolone pulse with tapering down and azathioprine treatment.	Methylprednisolone pulse with tapering down rituximab treatment.	Methylprednisolone pulse with tapering down and azathioprine treatment.	Methylprednisolone pulse with tapering down and azathioprine treatment.	Methylprednisolone pulse with tapering down with cyclophosphamide
Response	Remission	Partial remission	Partial remission	Poor compliance	Partial remission

Clinical presentations varied and included one or more of the following subacute unilateral weakness (*n* = 2) or cognitive impairment mainly in memory and executive function domains and gait abnormality (*n* = 1), subacute headache and visual disturbance (*n* = 1), and epileptic seizures (*n* = 1). All patients reported progressive holocephalic headaches and non-specific cognitive changes with overall progressive symptoms. Initial neurological examinations revealed abnormalities in all patients and included one or more of the following: cognitive or visual impairment of hemifield anopsia (*n* = 2), seizures with semiology of focal right facial spasm onset, and secondary generalization. EEG showing left-side slow waves in frontoparietal regions (*n* = 1) and unilateral upper motor neuron (UMN) signs (*n* = 2) were observed, characterized by right upper and lower limb weakness, brisk reflexes, and extensor responses at the initial neurological examination, all but one patient required walking aids, including (cane 2, wheelchair 1, bedridden 1). Patients’ serological evaluation ruled out other causes of their symptoms detailed in the methods above. Three patients underwent TEE or TTE, which were normal.

### Imaging and CSF studies

**Figure 1:**
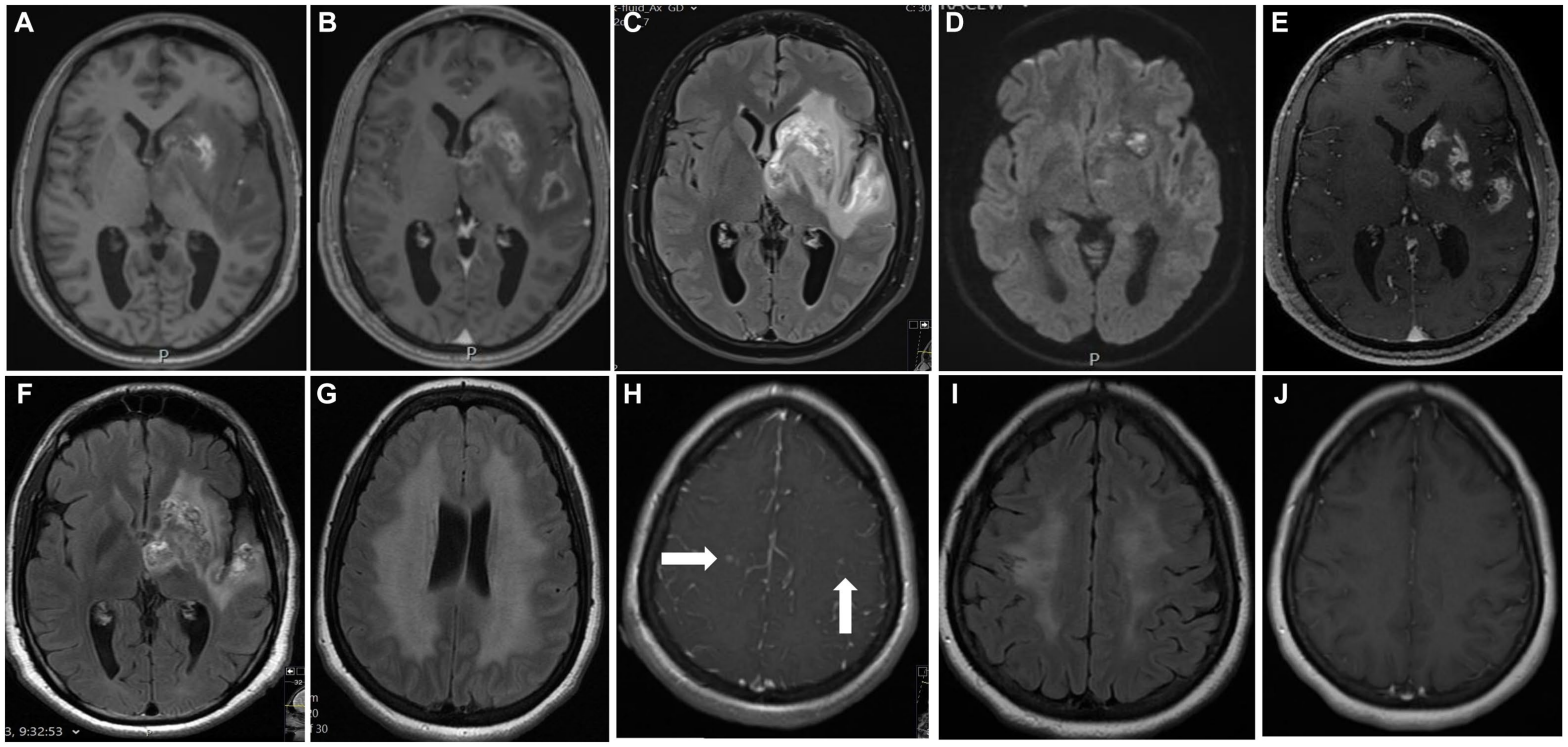
Main MRI features: Shown are selected figures for 2 of our 5 cases. The first case performed an MRI of the brain on admission **(A–D)** shows lesion with high signal on T1 weighted image at the basal ganglia **(A)** demonstrated areas of hemorrhage, with ring enhancement after gadolinium administration **(B)**, vasogenic edema at the temporal lobe on T2FLAIR **(C)** and restricted diffusion on DWI **(D)** at both left basal ganglia and insular cortex. **(E,F)** demonstrate the MRI during treatment with decrease degree on enhancement **(E)** and vasogenic edema **(F)**. Second case performed MRI on admission show symmetrical high signal on T2-FLAIR of the white matter at the corona radiata on both sides **(G)** with small foci of enhancement after gadolinium administration at the right centrum semiovale (**H**, arrows), and symmetrical areas of restricted diffusion on DWI of the centrum semiovle on both sides (NOT SHOWN). A consequent MRI after treatment show decrement of the high signal on T2-FLAIR **(I)**, and no enhancement after gadolinium administration **(J)**.

All patients underwent CTA (CT angiography) and cerebral angiography (*n* = 2), which were interpreted as with no abnormality. PET-CT (*n* = 2) showed no hypermetabolic areas. MRI imaging of the brain was performed on all patients and was abnormal in all of them (Figure 1). Abnormalities included one or more of the following abnormal signals in the basal ganglia, corona radiata, parietal lobe, and frontal lobe. These lesions demonstrated a range of imaging characteristics, including hemorrhage, vasogenic edema, restricted diffusion, and enhancement following gadolinium administration. Three patients demonstrated unilateral involvement with T2 hyperintensity and vasogenic edema surrounding the lesions, resulting in a local mass effect on fissures, gyrus, and ventricles (Figures 1A–F). On the other hand, two patients showed more confluent periventricular involvement with T2 hyperintensity and no enhancement after gadolinium administration or mass effect (Figure 1). Brain digital subtraction angiography was performed in two suspected cases based on MRI findings and excluded large or medium blood vessel abnormalities.

CSF analyses were conducted in three of the five patients (60%) and were contraindicated in two patients due to mass effect and a high risk for herniation; two of the three patients (66%) had lymphocytic pleocytosis with a median number of cells of 15 (range: 6–234). The median CSF protein level was 57 mg/dL (range: 41–84 mg/dL). CSF culture results, PCR testing for HSV1, HSV2, enterovirus and VZV, serology testing for WNV and syphilis, and cryptococcal antigen testing were all normal.

### Histopathological changes

**Figure 2:**
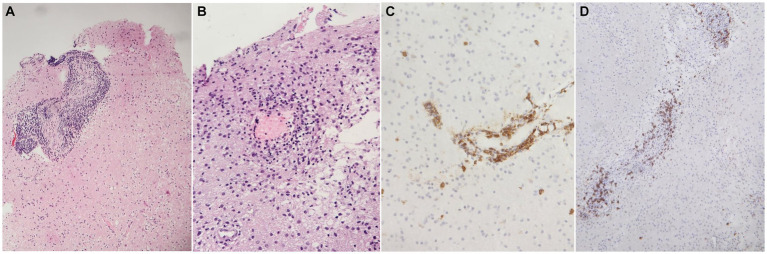
Main histopathological findings. **(A)** Brain tissue showing blood vessels massively infiltrated by small lymphocytes (H&E stain, 100x), **(B)** brain tissue showing a small blood vessel with necrosis of the wall and micro thrombus (H&E stain, 200X), **(C)** blood vessel wall massively infiltrated by CD3 positive T-lymphocytes (immunoperoxidase, 200x), **(D)** blood vessel wall infiltrated by CD20 positive B-lymphocytes (immunoperoxidase, 200x).

Brain tissue biopsies were performed in all five patients (re-examined by G.M). The pathological histological images of one patient were obtained and could not be retrieved, with only the report being available. The main pathological findings included massive infiltration of small and medium size blood vessels by small lymphocytes (Figure 2A). Additionally, blood vessels with microthrombi and necrosis of the vessel wall were observed (Figure 2B). Immunohistochemical stains were performed on all biopsies, revealing massive infiltration of the blood vessel walls by CD3-positive T-lymphocytes, as shown in Figure 2C, and CD20-positive B-lymphocytes, as shown in Figure 2D, in four patients. One patient had perivascular lymphoplasmacytoid infiltration with epithelioid granulomas with multinucleated giant cells, which was consistent with granulomatous CNS vasculitis. Stains for acid-fast bacilli and fungi were negative, and neoplastic lymphocytes were not found.

### Clinical characteristics, immunotherapy, and outcomes

All patients were treated with immunotherapy as indicated in Table 2. Initial treatment involved a course of intravenous methylprednisolone followed by a tapering regimen of oral prednisone in all patients. Two patients receive 500 mg of intravenous methylprednisolone for 5 days, followed by 60 mg of oral prednisone. Three patients received 1 g for 3 days, 500 mg for 2 days, and 60 mg of oral prednisone. Other concurrent treatments included azathioprine (*n* = 3), cyclophosphamide (*n* = 1), and rituximab (*n* = 1). Although most literature for PCNSV uses cyclophosphamide, we used a protocol that, in our department, showed promising results for vasculitis that is not PCNSV and had fewer side effects. Following treatment initiation, all patients demonstrated significant clinical and radiological improvement, as illustrated in Table 2. Notably, there was a substantial decrease in dependence on assistance for daily tasks, indicative of enhanced motor and cognitive abilities.

The median follow-up time was 56 months (range: 20–161 months). Patients were stable (*n* = 2), deteriorated (*n* = 1), or improved (*n* = 2). Deterioration was evident radiographically 2 years after receiving chronic low doses of prednisone and azathioprine, which led to the addition of rituximab. Rituximab treatment stopped the deterioration of the patient’s cognitive condition and significantly improved his neurologic condition, which allowed him to complete his higher education in later years; two of the patients still required ambulatory aids, with one using a cane and the other using a wheelchair, while the remaining three did not require any ambulatory aids.

The rituximab-treated patient is fully ambulatory, the cyclophosphamide-treated patient remained in a wheelchair, and two of the three patients treated with a combination of prednisone and azathioprine are fully ambulatory, while one uses a cane.

## Discussion

We report five cases of PCNS vasculitis with a median follow-up time of 56 months. We had a higher rate of male individuals (4:1 male predominance) than reported, with a 2:1 male-to-female ratio. The median age of onset was 31 years, significantly younger than the reported median age of 50 (2, 7), the last two findings are in accordance with a recently published large cohort in India (15). All patients clinically exhibited holocephalic headaches and progressively worsening neurological symptoms. The time from symptom onset to diagnosis was notably extended, with one case taking up to 4 years. This delay is attributed to the initial presentation of non-specific and non-localizing symptoms. In a particular case where the patient exhibited seizures and headaches, reversible cerebral vasoconstriction syndrome (RCVS) was initially suspected. However, the absence of a thunderclap headache led us to perform a brain biopsy, which ultimately confirmed the diagnosis of primary central nervous system vasculitis (PCNSV). The diagnostic consideration of RCVS is crucial due to its clinical and radiological similarities, particularly concerning headaches and MRI findings. Formerly RCVS was described as a subtype of PACNS, called benign angiopathy of the CNS (16).

From the diagnostic perspective, vasculitis can potentially be present at any age probably due to non-specific environmental triggers combined with genetic predisposition (Table 3). Most of our patients (4/5) were Muslim Arabs. The high prevalence of Muslim Arabs in our cohort could be attributed to selection bias, given that our hospital serves as a referral center for the northern part of Israel, where there is a higher proportion of Arab citizens than the central region. However, this prevalence still surpasses the proportion of Muslim Arabs in the northern population, which constitutes less than 80%. This observation may indicate a potential predisposition among this population group for autoimmune vasculitis (17). Solitary brain mass lesion was the imaging abnormality in 60% of our cases, while it has been reported that only 5–15% of histopathology-proven PACNS present with this imaging phenotype (12, 18–20), and only a few cases have been reported in the literature (11, 20–22). This highlights the importance of considering PACNS in the differential diagnosis of solitary lesions and gadolinium enhancement with atypical features. Using digital subtraction angiography on MRI was reported to show changes related to vasculitis in 50 to 90% of cases (23–25). This was performed in two of our patients but was unrevealing.

**Table 3:** Differential diagnosis of PCNSV.

	PCNSV	Autoimmune systemic vasculitis	RCVS	Moya Moya	Infectious vasculitis
Clinical presentation	Headache. Seizures. Cognitive changes. Focal neurological signs.	Headache. Seizures. Cognitive changes. Focal neurological signs. Systemic signs such as fatigue, asthma, and sinusitis.	Female patients > male patients. Thunderclap headache with photophobia and phonophobia. Focal neurological deficit. Seizures, confusion. History of migraine. Triptans use. Marijuana use. SSRIs treatment.	Headache. Seizures. Vision problems. Focal neurological signs	Fever, seizure, focal neurological signs. Systemic signs.
Brain MRI	Hemorrhagic/ischemic stroke and microangiopathy. White matter changes.	Hemorrhagic/ischemic stroke and microangiopathy. White matter changes.	Vasogenic edema and/or sulcal hyperintensities. Flair hyperintense vessels and subarachnoid hemorrhage.	Bilateral concentric luminal narrowing	Ischemic stroke. White matter changes.
Histologically	More robust lymphocytic and macrophage infiltration	Fibrinoid necrosis of blood vessel wall, granulocyte, and lymphocytic infiltration	No morphological changes	No morphological changes	More robust granulocytic infiltration
Treatment	Steroid, steroid sparring agent	Steroid, steroid-sparring agent	Blood pressure monitoring. Calcium channel blockers, preferably Verapamil.	Anti-platelets, calcium channel blockers angiography, and stenting in certain cases.	Antibiotics. Anti-parasitic. Anti-Fungal. Anti-Viral.

PCNS vasculitis is usually diagnosed by a combination of clinical and radio-pathological findings. CNS biopsy is considered the gold standard (1), and it was conducted in all our patients as part of the inclusion criteria for the study. Many other reports used various other criteria for diagnosis with the highest rate of histopathologically proven cases being 90% (15). The biopsy revealed blood vessels massively infiltrated by small lymphocytes similar to what is known in the literature (15, 26), while the granuloma-like subtype was the most common in the 1992 Mayo cohort and a more recent Mayo cohort (14, 27).

In our cohort, most patients (80%) were treated with steroids in combination with azathioprine with good outcomes having clinical–radiological improvement, and achieving prolonged remission (absence of relapse at ≥12 months after diagnosis). As this is a rare disease and RCTs are lacking, treatment for PCNSV is guided by expert opinion and retrospective data mostly. In our cohort, we used azathioprine to induce remission sparing the nephrological toxicity and fertility concerns among these patients, who are men and women of childbearing age (28). Clinical improvement was preserved in 40% of our patients. One patient remained on low-dose prednisone treatment (10 mg) with residual cortical blindness, and another one was on low-dose prednisone combined with full-dose azathioprine and antiseizure drugs after 9 and 13 years of follow-up, respectively. Radiological relapse was demonstrated in one patient on steroids and azathioprine treatment for 2 years; he needed low-dose azathioprine due to anemia that developed while having full-dose treatment. The patient’s treatment was switched to rituximab, resulting in a favorable clinical outcome. Initially, the patient was maintained on a low dose of azathioprine for a year. Following this period, a radiological relapse was observed, prompting the transition to rituximab treatment.

Our study is unique in presenting a collection of atypical cases, which needs to increase awareness of this diagnosis. Differential diagnosis in these cases was a demyelinating lesion or tumor, which is why all patients had a brain tissue biopsy. There are a few limitations to our study. Its retrospective nature binds it to possible selection bias and shortcomings of data collection. Some of the imaging studies were performed on different scanners with variable strength and heterogeneous protocols.

In this study, we present a cohort of PCNSV patients exhibiting focal neurological signs alongside mass-like brain lesions on MRI imaging. It may represent an intense inflammation process. The challenges of delayed diagnosis and the diverse range of neurological manifestations, encompassing cognitive disturbances and seizures, highlight the intricate nature of PCNSV. The persistence of progressive headaches and cognitive changes among patients underscores the diagnostic complexities. Histopathological analysis through brain biopsies revealed significant lymphocytic infiltration, thrombi, and necrosis within the PCNSV patient group. Effective immunotherapy yielded substantial improvements in clinical and radiological outcomes. Throughout the 56-month median follow-up, outcomes varied, encompassing stability, deterioration, or improvement. The advantage of rituximab in deteriorating cases is that it has the potential to reverse and stop the deterioration from going further. Notably, one patient achieved cognitive normalcy and was able to advance their education.

## Data availability statement

The original contributions presented in the study are included in the article/supplementary material, further inquiries can be directed to the corresponding author.

## Ethics statement

The studies involving humans were approved by Rambam Healthcare campus Haifa Israel. The studies were conducted in accordance with the local legislation and institutional requirements. Written informed consent for participation was not required from the participants or the participants’ legal guardians/next of kin because only retrospective collecting of data.

## Author contributions

TS: Writing – original draft, Data curation. AR: Writing – original draft, Data curation. GM: Writing – review & editing, Data curation. AS: Writing – review & editing. PS: Writing – review & editing, Data curation. SS: Writing – original draft, Conceptualization.
